# The clinical effectiveness of using a predictive algorithm to guide antidepressant treatment in primary care (PReDicT): an open-label, randomised controlled trial

**DOI:** 10.1038/s41386-021-00981-z

**Published:** 2021-02-26

**Authors:** Michael Browning, Amy C. Bilderbeck, Rebecca Dias, Colin T. Dourish, Jonathan Kingslake, Jürgen Deckert, Guy M. Goodwin, Philip Gorwood, Boliang Guo, Catherine J. Harmer, Richard Morriss, Andreas Reif, Henricus G. Ruhe, Anneke van Schaik, Judit Simon, Victor Perez Sola, Dick J. Veltman, Matilde Elices, Anne G. Lever, Andreas Menke, Elisabetta Scanferla, Michael Stäblein, Gerard R. Dawson

**Affiliations:** 1 P1vital Ltd, Howbery Park, Wallingford, UK; 2 Department of Psychiatry, University of Oxford, Oxford, UK; 3 Oxford Health NHS Foundation Trust, Warneford Hospital, Oxford, UK; 4 P1vital Products Ltd, Howbery Park, Wallingford UK; 5 Department of Psychiatry, Psychosomatics and Psychotherapy, Center of Mental Health, University Hospital of Würzburg, Würzburg, Germany; 6 Université de Paris, Institute of Psychiatry and Neuroscience of Paris (IPNP), INSERM U1266, Paris, France; 7 GHU Paris Psychiatrie et Neurosciences, Hôpital Sainte Anne, Paris, France; 8 Division of Psychiatry and Applied Psychology, University of Nottingham, Nottingham, UK; 9 Department of Psychiatry, Psychosomatic Medicine and Psychotherapy, University Hospital Frankfurt - Goethe University, Frankfurt am Main, Germany; 10 Department of Psychiatry, Radboudumc, Nijmegen, Nijmegen, The Netherlands; 11 Donders Institute for Brain, Cognition and Behavior, Radboud University, Nijmegen, The Netherlands; 12 Department of Psychiatry, Amsterdam Public Health Research Institute, Amsterdam UMC, Amsterdam, The Netherlands; 13 Department of Health Economics, Center for Public Health, Medical University of Vienna, Vienna, Austria; 14 Hospital del Mar Medical Research Institute, IMIM, Barcelona, Spain. Centro de Investigación Biomédica en Red (CIBERSAM), Madrid, Spain; 15 Department of Psychiatry, Amsterdam UMC, Amsterdam Neuroscience, Amsterdam, The Netherlands; 16 Medical Park Chiemseeblick, Bernau-Felden, Germany; 17 Université Paris, ED 450, Paris, France

**Keywords:** Depression, Human behaviour

## Abstract

Depressed patients often do not respond to the first antidepressant prescribed, resulting in sequential trials of different medications. Personalised medicine offers a means of reducing this delay; however, the clinical effectiveness of personalised approaches to antidepressant treatment has not previously been tested. We assessed the clinical effectiveness of using a predictive algorithm, based on behavioural tests of affective cognition and subjective symptoms, to guide antidepressant treatment. We conducted a multicentre, open-label, randomised controlled trial in 913 medication-free depressed patients. Patients were randomly assigned to have their antidepressant treatment guided by a predictive algorithm or treatment as usual (TaU). The primary outcome was the response of depression symptoms, defined as a 50% or greater reduction in baseline score of the QIDS-SR-16 scale, at week 8. Additional prespecified outcomes included symptoms of anxiety at week 8, and symptoms of depression and functional outcome at weeks 8, 24 and 48. The response rate of depressive symptoms at week 8 in the PReDicT (55.9%) and TaU (51.8%) arms did not differ significantly (odds ratio: 1.18 (95% CI: 0.89–1.56), *P* = 0.25). However, there was a significantly greater reduction of anxiety in week 8 and a greater improvement in functional outcome at week 24 in the PReDicT arm. Use of the PReDicT test did not increase the rate of response to antidepressant treatment estimated by depressive symptoms but did improve symptoms of anxiety at week 8 and functional outcome at week 24. Our findings indicate that personalisation of antidepressant treatment may improve outcomes in depressed patients.

## Introduction

Depression, usually accompanied by anxiety, is the leading cause of years lived with disability worldwide [1]. It results in a marked functional impairment of patients and, consequently, a huge economic impact [2]. While a large number of medications have been found to reduce symptoms of depression [3], as many as 50% of patients do not respond to the initial medication prescribed [4] and require sequential trials of different treatments. Further, appreciable subjective improvement is often only apparent after four to six weeks of treatment, prolonging the duration of each treatment trial [5, 6]. In practice, the delay between trials of different treatments is longer than this [7]. As a result, there is often a significant delay between the decision that a patient requires treatment and starting that patient on an effective antidepressant.

Personalised medicine, using the characteristics of a patient to select the most effective treatment, offers one route by which this delay may be reduced [8]. A number of demographic, clinical, cognitive and physiological measures, collected either at baseline [9–14] or after a brief initial period of treatment [15–17], have been reported to predict clinical response. However, the crucial next step in the development of a personalised approach to treatment, which has yet to be taken for drugs for depression, is to test whether using these factors to guide treatment selection has a clinically meaningful impact on outcomes [8].

In previous work, we have described the development and validation of a predictive algorithm (the PReDicT test) based on measures of affective processing bias and symptoms of depression [15]. Affective processing bias describes the tendency for individuals to preferentially remember, interpret or pay attention to positive relative to negative information [18]. Depressed patients, for example, display a negative bias, interpreting ambiguous facial expressions as being less happy than non-depressed participants [18]. Antidepressant medications act very early in the course of treatment to induce a positive processing bias [18–20]. This early cognitive effect is seen across different classes of antidepressant [21] and is associated with improved treatment response [22]. In our classifier development work, we used measures of affective processing and symptoms of depression after one week of antidepressant treatment to predict response at 6 weeks with an accuracy of 60% [15].

In the PReDicT trial [23], we tested the clinical effectiveness of using the PReDicT algorithm to guide treatment versus unguided care in a large sample of patients presenting with symptoms of depression and anxiety. Recruitment was predominately from primary care in five European healthcare systems. Patients were randomised to have treatment guided by the algorithm, which could prompt the clinician to make early changes in treatment [24], or to treatment as usual (TaU), and were followed up for a year after randomisation.

## Patients and methods

### Study design and patients

The PReDicT trial was a two-arm, multisite, open-label, randomised controlled trial of a medical device (the PReDicT test) in patients from five European countries (UK, Spain, Germany, France and the Netherlands, see Supplementary Materials for more information on the recruitment centres) [23]. Patients were eligible for inclusion if they were aged between 18 and 70 and were deemed by their treating clinician to require initiation of treatment with a selective serotonin reuptake inhibitor (SSRI, excluding fluoxetine, due to its longer half-life) for the treatment of a depressive episode. Exclusion criteria included current treatment with an antidepressant, a previous history of mania or a presentation that required immediate referral to a separate service (e.g., significant suicidal intent requiring enhanced care).

All patients provided written informed consent. Ethics approval was obtained from the National Research Ethics Service committee, North East York (16/NE/0095), Ile de France Ethics Committee (MDPT-RIAL/MM/2016-AO1054-47), Medisch Ethische Toetsingcommissie VU Medisch Centrum (2016.294 NL58027.029.16), CEIC Par de Salut Mar (2016/6795/I), Ethik Komission der Universitat Wurzburg (117/16-sc) and Ethik Kommission des Fachbereichs Medizin, Universitatsklinikum der Goethe Universitat Frankfurt (34/17B).

### Randomisation and masking

Patients were randomised to have antidepressant treatment guided by the PReDicT test (PReDicT group) or to receive treatment as usual (TaU group) using a 1:1 ratio across the study. Following recruitment by the treating clinician at study sites, a study researcher registered each patient onto the online electronic patient-reported outcome system (P1vital^®^ ePRO system) that performed the randomisation. Randomisation was stratified by study country and minimised by (a) gender (male/female), (b) age (18–44 and 45–70) and (c) baseline depression severity (mild/moderate, defined as a score of ≤15, and severe/very severe, defined as a score of >15) using the standardised severity cut-offs from the 16 items, self-report version of the Quick Inventory of Depressive Symptoms (QIDS-SR-16) questionnaire [25]. Patients were not informed of the group they had been randomised into but could deduce this from the information they received during the study (only patients in the PReDicT arm would be asked to change medication based on the results of their PReDicT test). The treating clinician and raters for the Montgomery–Åsberg Depression Rating Scale (MADRS) [26] were aware of patients’ randomisation group for the same reason. The trial statistician was blinded to group allocation. This is therefore an open-label study. All treatment decisions were made by the treating clinician, not by study researchers.

The first 110 patients were assigned to the PReDicT arm or the TaU arm in a 1:10 ratio. During this phase, minimisation was not used. The first 67 patients recruited into the TaU arm were used to refine the predictive algorithm (N.B. clinical response at week 8 was required to retrain the algorithm and this was available at the time for only 67 of the 100 patients randomised to the TaU arm). The rationale for this was that the algorithm had previously been trained using data from UK-based patients who had only received citalopram, raising the possibility that it was not representative of data from this study in which patients were recruited from across Europe and received a range of treatments. In order to address this concern, the algorithm was retrained (see below for details) using more representative participants from the TaU arm. NB the primary analysis is reported for all patients, with a sensitivity analysis reported only for those patients recruited following the algorithm update in the supplementary results.

### Procedures

Following recruitment, patients were prescribed an SSRI (excluding fluoxetine) by their treating clinician but did not initiate the treatment until the baseline assessment was completed. At the time of prescription, the treating clinician was not aware of the patients’ randomisation group. The baseline assessment took place within 7 days of antidepressant prescription and consisted of self-reported questionnaires and the affective processing task, both administered by the ePRO system, and an observer-rated assessment of depressive symptoms. The affective processing task [27] consisted of pictures of faces displaying a range of emotional expressions (sad, happy, fearful, surprise, disgust, anger and neutral), at ten different intensities (10–100% in steps of 10%) that were displayed for 500 ms each. Patients were asked to categorise the expression of the faces as one of the emotions listed above. The predictive algorithm was based on changes in the performance of the affective processing task as well as individual item scores from the QIDS-SR-16 questionnaire between the baseline session and weeks 1 and 2. Patients were asked to initiate their prescribed treatment, and report this on the ePRO system, after completion of the baseline session.

All patients repeated the affective processing task and QIDS-SR-16 self-report questionnaire 1 week (7–9 days) after treatment initiation. At this point, the treating clinicians of patients in the PReDicT arm were informed of the result of the PReDicT test and, if the prediction was non-response, were advised to adjust the patient’s antidepressant treatment. Where patietns were predicted to be responding, clinicians were advised not to alter treatment. Clinicians were encouraged to come to a collaborative decision on prescribing based on the result of the PReDicT test and other clinically relevant information, e.g., side effects and patient preferences. For patients in the TaU arm, clinicians did not receive the results of the PReDicT test and were asked to manage patients as per normal practice (i.e., alter treatment in response to lack of efficacy and/or side effects). Those patients in the PReDicT arm predicted not to be responding repeated the PReDicT test at week 2 (a further 7–9 days), with the result of the prediction again provided to their clinician. Thereafter, the PReDicT test was not administered again and all patients were treated according to local prescribing guidelines. All patients were asked to complete the QIDS-SR-16 weekly, using the ePRO system until week 8, at which point patients attended the study centre to repeat the baseline assessments (other than the affective processing task). Following this, patients completed remote assessments using the ePRO system of the QIDS-SR-16 monthly to one year and the SAS-screener, the 14-item “screener” form from the social adjustment scale (SAS scale [28]), at months 6 and 12. Adverse events were collected during study visits (until week 8). Adverse events were not collected during the remote follow-up phase.

The PReDicT test used a support vector machine [29] to provide a binary prediction (patient responding/not responding to treatment) based on the change of depressive symptoms (measured using the QIDS-SR-16) and change in the performance of the effective bias task over the first one and 2 weeks of treatment. A detailed description of the development of the test is provided here [15]. In brief, the algorithm used in the current study (including the refinement step after 67 patients had been recruited) selected the top 50% predictive features derived from changes in the face-processing task and the QIDS-SR-16, with the C-parameter selection based on the leave-one-out accuracy within the training sample. Data from a subset of patients in the TaU arm were used to provide an out-of-sample accuracy in the report of the classifier development [15], with no other overlap between study participants.

Clinicians were asked to use antidepressants and doses that were consistent with local prescribing guidelines. In response to predictions of non-response, clinicians were asked to consider either (a) increasing the dose of the antidepressant, (b) changing the antidepressant or (c) augmenting the antidepressant (e.g., adding mirtazapine to ongoing treatment with an SSRI).

### Outcomes

The primary outcome was treatment response at week 8. The response was defined as a 50% or greater reduction of the baseline QIDS-SR-16 score. Additional prespecified outcomes were; change in anxiety scores at week 8 (measured using the Generalised Anxiety Disorder Assessment, seven-item version, GAD-7 [30]), remission of depression at week 8 (defined as QIDS-SR-16 score of ≤ 5), change in the individual item scores from the QIDS-SR-16 measuring restlessness and sadness at week 8, change in symptoms of depression (treated as a continuous variable) across 12 months (measured using QIDS-SR-16), change in observer- reported symptoms of depression (treated as dichotomous response and as a continuous variable and measured using the MADRS at week 8 and change in functional outcome across 12 months (measured using the SAS-screener). Patients also completed detailed health economic, acceptability and cognitive functioning measures that will be reported separately [23].

### Statistical analysis

The sample size was determined based on a minimum clinically relevant effect size (i.e., the difference in effect size between the TaU and PReDicT arms), which was set at 10% for the primary outcome. Setting alpha (two-tailed) at 0.05 and power at 80% with the estimated baseline response rate of 40% [15] indicated that a total sample size of 776 participants with primary outcome data (388 per group) would be required. The estimated attrition rate for the study was 35%, suggesting a total recruitment target of 1200. In practice, attrition was substantially lower (15%) than expected, meaning that the target sample was achieved following the recruitment of 913 patients.

Analyses and reporting were in line with CONSORT guidelines and were described in a pre-published protocol [23] and statistical analysis plan (SAP, DOI: 10.5281/zenodo.1235968). Efficacy analyses and outcomes were defined in the protocol and SAP and were not changed during the course of the study.

Intention-to-treat analyses were used, the population analysed was all participants randomised to the trial. The primary analysis used multilevel logistic regression with age, gender and baseline depression score included as covariates and country as higher-level units to quantify the effect of group membership on treatment response as an odds ratio (OR) with 95% confidence interval. Missing outcomes were imputed by multiple imputations under the missing at random assumption. To determine the influence of ‘data missingness’ on the primary analysis, a sensitivity analysis was conducted using only the observed data. Additional categorical outcomes were analysed in a similar manner as the primary outcome. Continuous additional outcomes were analysed using a multilevel linear regression with categorical time and time x arm interactions included as additional covariates when there was more than one follow-up time point.

Non-prespecified exploratory mediation analysis is also presented. This analysis sought to test the potential mediating relationship between the observed differences between groups. Methodological details of this analysis are provided in the Supplementary Methods. The study was overseen by an independent data-monitoring committee. All analyses were performed in STATA version 16. The study was registered with clinicaltrials.gov, reference NCT02790970 before study commencement, with no significant changes to the methodology being made during the course of the study.

## Results

**Fig. 1:**
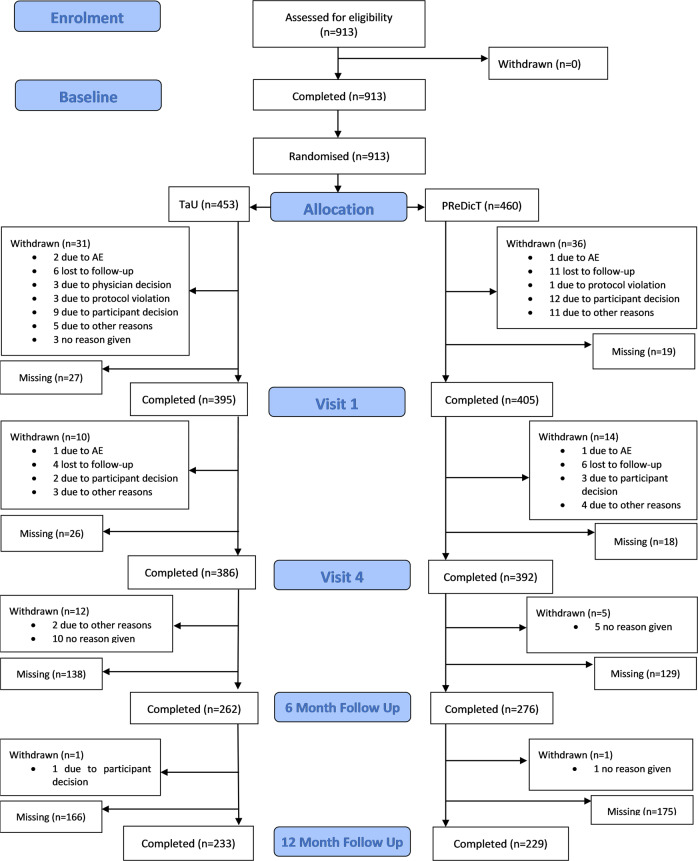
Consort diagram for the study. Patient data listed as missing indicate that data were not collected for a specific time point, although the patient remained in the study. Withdrawn patients did not provide further study data.

Between July 26, 2016 and September 28, 2018, we recruited and randomised 913 patients, 460 (50%) to the PReDicT arm and 453 (50%) to the TaU arm (Fig. 1). The trial achieved its recruitment target with complete data for the primary analysis available for 778 patients (PReDicT 392 patients, TaU 386 patients). Approximately half were recruited in the United Kingdom, with the remainder recruited throughout the other European countries. A detailed summary of recruitment and retention by country is provided in the supplementary materials.

The baseline characteristics were similar between groups (Table 1). Recruited participants were largely white (90%), with more females (62%) than males (38%), reflecting the usual 2:1 gender ratio in depression. The mean age was approximately 40 years, with the age range spanning from the minimum to maximum permitted (18–70). The mean QIDS-SR-16 score was 15, on the borderline between moderate and severe depression [25]. The mean anxiety score was 13.5 (GAD-7), similar to previous outpatient clinical samples [31], and functional level scores were 63 (SAS-SR screener), which is also similar to previous samples of depressed primary care patients [28].

**Table 1:** Baseline demographic and clinical details of patients.

		PReDicT arm (*n* = 460)	TaU arm (*n* = 453)
*Country,* n *(%)*			
	France	39 (8%)	37 (8%)
	Germany	63 (15%)	67 (14%)
	Spain	82 (18%)	82 (18%)
	The Netherlands	28 (6%)	26 (6%)
	UK	248 (53%)	241 (54%)
Age mean (SD)		38.7 (13.53)	39.21 (14)
*Sex,* n *(%)*			
	Female	285 (62%)	282 (62%)
	Male	175 (38%)	171 (38%)
Ethnicity*, n *(%)*			
	White	374 (90%)	374 (89%)
	Ethnic minority	47 (10%)	42 (11%)
Years of education mean (SD)		14.14 (3.66)	14.10 (3.47)
*Recruited from primary or secondary care*			
	Primary	359 (78%)	345 (76%)
	Secondary	101 (22%)	108 (24%)
*Employment status*			
	Full or part-time work or student	165 (36%)	152 (34%)
	Unemployed, sickness leave or retired	295 (64%)	301 (66%)
*Living situation*			
	Living with a spouse or partner	265 (58%)	244 (54%)
	Not living with a spouse or partner	195 (42%)	209 (46%)
*Present or past relationships*			
	Ever been married, lived with a partner or had children	319 (69%)	318 (70%)
	Never married, lived with a partner or had children	141 (31%)	135 (30%)
*Family history of depression,* n *(%)*			
	No	225 (49%)	215 (47%)
	Yes	235 (51%)	238 (53%)
QIDS-SR-16 mean (SD)		15.67 (4.53)	15.65 (4.19)
MADRS mean (SD)		28.04 (7.45)	28.07 (7.08)
GAD-7 mean (SD)		13.59 (4.92)	13.77 (4.85)
SAS-SR mean (SD)		63.06 (11.16)	62.88 (11.28)

*A local ethical requirement prevented the collection of data on ethnicity from patients in France. QIDS-SR-16; Quick Inventory of Depressive Symptoms, 16-item self-report version (score range 0–27). MADRS; Montgomery–Åsberg Depression Rating Scale (score range 0–60). GAD-7; Generalised Anxiety Disorder Assessment, seven-item version (score range 0–21). SAS-SR; Social Adjustment Scale, self-report screener form, T-score (note a higher score indicates greater impairment, score range 38–90). Scales are reported as mean (SD).

Patient retention for the primary outcome at week 8 was 85%. Retention at the end of follow-up (month 12) was 50%, with no statistical difference between groups for either figure. Clinician behaviour, in terms of prescribed medication, was influenced by the results of the PReDicT test in the PReDicT arm with 65% of patients, who were predicted not to be responding, having their medication altered within the first 2 weeks, compared to 15% who were predicted to be responding (20 and 16%, respectively, in the TaU group, see Supplementary Materials for more details).

Response rates estimated with QIDS-SR-16 at week 8 were 55.9% (95% CI: 48.52–63.05) for the PReDicT arm and 51.8 (95% CI: 44.44–59.08) for the TaU arm. After adjustment for baseline scores and stratification variables, the odds ratio of the difference between arms was not significant at 1.18 (95% CI: 0.89, 1.56), *P* = 0.250 (Table 2). There was no difference in rates of remission of depressive symptoms using either the QIDS-SR-16 or the MADRS scales or in the QIDS-SR-16 items measuring sadness and restlessness (Table 2).

**Table 2:** Analyses of measures of depressive symptoms.

		PReDicT group		TaU group			
Categorical outcomes							
	Time point	*N*	% (95% CI)	*N*	% (95% CI)	Odds ratio (95% CI)	*P* value
QIDS-SR-16 response (primary outcome)	**Week 8**	**392**	**55.91 (48.52, 63.05)**	**389**	**51.8 (44.44, 59.08)**	**1.18 (0.89, 1.56)**	**0.250**
QIDS-SR-16 remission	Week 8	392	35.17 (27.69, 43.45)	389	36.16 (28.49, 44.61)	0.96 (0.69, 1.32)	0.792
MADRS response	Week 8	378	57.61 (51.47, 63.53)	372	58.44 (52.32, 64.31)	0.97 (0.73, 1.28)	0.812
MADRS remission	Week 8	378	29.03 (23.88, 34.78)	372	28.56 (23.48, 34.23)	1.02 (0.76, 1.38)	0.880

Continuous outcomes							
	Time point	*N*	Mean change (95% CI)	*N*	Mean change (95% CI)	Difference between groups (95% CI)	*P* value
QIDS-SR-16 sadness item	Week 8	392	−1.34 (−1.46, −1.23)	389	−1.28 (−1.40, −1.16)	0.06 (−0.06, 0.18)	0.295
QIDS-SR-16 restlessness item	Week 8	392	−0.53 (−0.62, −0.43)	389	−0.44 (−0.54, −0.34)	0.09 (−0.03, 0.20)	0.133
MADRS	Week 8	378	−14.55 (−15.57, −13.54)	372	−14.46 (−15.49, −13.43)	0.10 (−1.03, 1.23)	0.867
QIDS-SR-16							
	Week 8	392	−7.70 (−8.51, −6.89)	389	−7.30 (−8.12, −6.89)	0.4 (−0.28, 1.07)	0.250
	Week 24	276	−8.10 (−8.94, −7.26)	262	−7.76 (−8.59, −7.26)	0.29 (−0.4, 0.99)	0.361
	Week 48	229	−8.95 (−9.77, −8.13)	233	−8.71 (−9.59, −7.83)	0.24 (−0.54, 1.02)	0.547

Primary outcome highlighted in bold. *QIDS-SR-16* quick inventory of depressive symptoms, 16-item self-report version, *MADRS*   Montgomery–Åsberg Depression Rating Scale. Cut off scores for remission= QIDS-SR-16 ≤ 5, MADRS ≤ 7.

We did find evidence of a greater reduction in symptoms of anxiety in the PReDicT arm than the TaU arm. There was also a significantly greater improvement in functional outcomes as measured using the SAS-SR screener at month 6, with the difference becoming non-significant by month 12 (Table 3).

**Table 3:** Analyses of measures other than symptoms of depression.

	PReDicT group		TaU group			
Time point	*N*	Mean change (95% CI)	*N*	Mean change (95% CI)	Difference between groups (95% CI)	*P* value
*GAD-7*						
**Week 8**	**391**	**−6.12 (−6.83, −5.41)**	**389**	**−5.44 (−6.14, −4.74)**	**0.68 (0.03, 1.32)**	**0.040**
*SAS-SR*						
Week 8	391	−7.60 (−8.57, −6.64)	389	−6.51 (−7.47, −5.54)	1.09 (−0.26, 2.45)	0.112
**Week 24**	**274**	**−9.70 (−10.79, −8.61)**	**258**	**−7.48 (−8.60, −6.36)**	**2.22 (0.74, 3.70)**	**0.004**
Week 48	228	−10.28 (−11.50, −9.05)	231	−9.60 (−10.81, −8.39)	0.68 (−0.73, 2.08)	0.346

Significant results highlighted in bold. *GAD-7* Generalised Anxiety Disorder Assessment, 7-item version, *SAS-SR* Social Adjustment Scale, self-report screener form, T-score (note a higher score indicates greater impairment).

### Exploratory analysis

The finding that patients in the PReDicT group had lower anxiety at week 8 and improved functional outcome at month 6 suggested that the improved functional outcome may have arisen due to the earlier effects on anxiety. We tested this possibility using a non-prespecified exploratory mediation analysis that demonstrated a significant mediation effect −0.47 (0.24), *P* = 0.048. Further details of this analysis are included in the Supplementary Materials.

We recorded 158 adverse events in the PReDicT group and 167 in the TaU group during the study; the most common type of event in both groups was gastrointestinal disorder. Of these events, two were judged to be potentially related to the PReDicT test (both were headaches after completing the test). In total, five serious adverse events were recorded in the PReDicT group and 8 in the TaU group, none of which were related to the PReDicT test. Three serious adverse events involved attempted suicide (all in the TaU group) and one hospitalisation because of increased suicidality (in TaU group). There were no deaths recorded.

## Discussion

In our primary analysis, we did not find an improved response of depressive symptoms to antidepressant treatment in the PReDicT group at week 8. Analyses of additional prespecified outcomes confirmed no improvement in recovery from depression, or depressive symptom level, but did show a greater reduction in symptoms of anxiety, at week 8 and of functional outcomes at week 24. In a post hoc exploratory analysis, there was evidence that the improvement in function at 24 weeks may have been mediated by improvement in anxiety symptoms at 8 weeks.

The effect on symptoms of anxiety and functional outcomes, but not on symptoms of depression is similar to the effects of the SSRI sertraline reported in a recent large independent trial in UK primary care patients [32]. Patients meeting the criteria for entry into either study were deemed to be depressed or in a depressive episode, but no severity threshold was applied. Both studies suggest that, in this group of primary care patients, the change in depressive symptoms is a relatively insensitive measure of the effect of SSRI treatment compared with anxiety or functional measures of outcome. Accordingly, depression symptoms may not have been the ideal target for our predictive algorithm. An algorithm trained to predict the change in symptoms of anxiety or functional recovery may be more useful and will be worth exploring in future. More generally, the utility of antidepressant medication is determined by the probability of beneficial and adverse outcomes, and it may be possible to combine predictive approaches that are separately sensitive to both. As an example, there is some early support for the use of pharmacogentic predictors of gene–drug interactions, which are thought to be linked to side effects, during initial antidepressant selection and dosing [33, 34]. Combining this approach with later predictive assays of treatment outcome, such as the PReDicT test described here, potentially allows an efficient method for selecting effective treatments while minimising side effects.

Patients understandably identify functional outcomes as particularly important measures of treatment success [35]. Overall, the current version of the PReDicT test did bring forward functional recovery in depressed patients and improved the subset of symptoms captured by the GAD-7 scale at week 8. The results of the exploratory mediation analysis suggest a potential mechanistic pathway for this result, with the initial change in symptoms of anxiety at week 8 significantly impacting the improvement in functional outcome at week 24. At the population level, anxiety disorders and particularly generalised anxiety are very commonly comorbid with depression [36]. Our finding that a reduction in symptoms of anxiety was associated with enhanced later functioning strengthens the case for targeting these symptoms during treatment. One caveat to this interpretation is that the effect of the intervention on symptoms of anxiety was less robust than its effect on functional improvement (see sensitivity analyses in Supplementary Materials) suggesting that a reduction of anxiety may be one of a number of mechanisms accounting for the functional improvement. The greater improvement in function at week 24 became non-significant by week 48; this was to be expected because the logic of using the PReDicT test is not that it enhances the underlying efficacy of antidepressant medication, but rather that it can facilitate more rapid identification and initiation of more effective treatment. Thus, use of the test is expected to result in an earlier response to treatment rather than increase its efficacy.

The motivation for developing personalised approaches to antidepressant treatment is the ability to rationally select the treatment that is individually most effective or has fewest side effects [8]. To date, studies in this area have sought to identify factors that are associated with future response to treatment [9–13, 15–17]. However, if personalised approaches are to be clinically useful, simply predicting the response to treatment is not sufficient. Rather, the prediction must influence clinician behaviour and, ultimately, patient outcome. The PReDicT study is, to our knowledge, the first robust assessment of this approach for antidepressant treatment and clearly showed the ability to influence clinician’s routine. Our finding, that use of the PReDicT test in primary care settings, across a range of healthcare systems improved anxiety and functional outcome, therefore, provides evidence that the selection of antidepressant medication may be improved in practice by deploying a personalised approach. While it will remain important to improve the predictive performance of treatment outcome classifiers, perhaps by combining demographic, cognitive and biological features [9–13], testing their clinical utility necessarily requires deployment in randomised designs such as those reported here.

A number of factors may have limited the overall effectiveness of the intervention tested in this study. First, the accuracy of the predictive algorithm was modest at 57.5% (see Supplementary Materials). It may be possible, and would clearly be desirable, to develop an algorithm with enhanced predictive properties, although it will be essential to test any such algorithm in prospective studies of the target clinical population, such as that reported here, rather than simply using samples of convenience as has previously been the norm. Indeed, data from the TaU arm of this study, which includes a substantial population of patients, across a number of healthcare systems, with high-quality cognitive, clinical and demographic data, may facilitate the development of an improved algorithm. Second, we focused on effectiveness rather than efficacy, requesting but not requiring clinicians to alter treatment in response to a prediction of non-response. The prediction that a patient was not responding only prompted a change of medication in 65% of cases (see Supplementary Materials), limiting the potential effectiveness of the intervention. Third, we did not specify how the treatment should be altered following a prediction of non-response, leaving it to the treating clinician to decide. As a result, changes in the dose of antidepressant were by far the commonest alteration made to treatment, rather than a switch to or augmentation of treatment with another drug (see Supplementary Materials). Given the doubt as to the efficacy of dose increases for common antidepressants [37, 38], this may have limited the impact of the intervention. Last, the overall response rate in the study was high, being about 10% higher than in our previous study of primary care patients in the United Kindom that did not involve weekly self-rating of symptoms by patients [15]. This raises the possibility that some aspect of study activity, such as the self-rating of symptoms or involvement of secondary care services (as occurred in some of the countries in this study), may have increased response rates in both groups, in effect adding an aspect of collaborative care to normal practice [39]. Qualitative data from acceptability and user experience interviews were collected during the study and will be reported separately. These data may shed light on how patients’ views of the algorithm influenced their response. Lastly, randomisation occurred at the level of the patient rather than the site and thus the TAU arm may have been influenced by behaviour learned in the active arm (to increase the dose or switch antidepressant, for example). All these factors may have served to weaken the contrast between the two arms of this study.

In summary, our finding that the use of a predictive algorithm to guide antidepressant treatment improves symptoms of anxiety and functional outcomes provides initial support for the use of personalised medicine approaches in the treatment of depression. This finding illustrates the potential benefit of developing the insight gained from mechanistic and experimental medicine studies of treatment mechanisms to build clinical tools that help patients.

## Funding and disclosure

This project received funding from the European Union’s Horizon 2020 research and innovation programme under grant agreement No 696802. This publication reflects only the authors’ views and neither the Horizon 2020 research and innovation programme nor the European Commission is responsible for any use that may be made of the information it contains. We thank the patients involved in the study and the staff of the participating clinical sites for their help in recruiting patients and running the study. We also thank those that have helped with the running of the study, in particular Lisa Pearce Collins, Sam Campbell, Garima Sharma, Mar Dziedzic and Hannah Alker. GMG is an Emeritus NIHR Senior Investigator. CJH is supported by the Oxford Health NIHR Biomedical Research Centre. The views expressed are those of the author(s) and not necessarily those of the NHS, the NIHR or the Department of Health. MB declares grants from the MRC and Wellcome Trust during the conduct of the study. He was employed by the trial CRO, P1vital Ltd, during the study and owns shares in P1vital Products Ltd (which owns the PReDicT algorithm and is the study sponsor). He has worked as a consultant for J&J and CHDR and has accepted travel funds from Lundbeck. GD, ACB are employees of P1vital Ltd. RD and JK are employees of P1vital Products Ltd. GD, JK, CTD own shares in P1vtial Ltd. JK, RD, CTD and GD own shares in P1vital Products Ltd. JD reports grants from the DFG, BMBF and Vogel Foundation during the conduct of the study. He is Co-PI with BioVariance in a study financed by the Bavarian Secretary of Commerce. GMG holds shares in P1vital and P1vital Products and has served as consultant, advisor or CME speaker in the last 3 years for Allergan, Angelini, Compass pathways, Evapharm, MSD, Janssen, Lundbeck Otsuka/Takeda, Medscape, Minerva, P1vital, Pfizer, Sage, Servier, Shire and Sun Pharma. PG has received, over the last 5 years, fees for presentations at congresses or participation in scientific boards from Alcediag-Alcen, Angelini, GSK, Janssen, Lundbeck, Otsuka, SAGE and Servier. CJH has received consultancy fees from P1vital Products Ltd as well as Janssen, Lundbeck, Sage Pharmaceuticals, Pfizer, Servier and Zongeixs. AR has received honoraria for lectures or advisory boards from Medice, Shire/Takeda, Janssen, SAGE, Servier and neuraxpharm. HGR has received speaking fees from Lundbeck. BG, RM, AvS, JS, VPS, DJV, ME, AGL, AM, ES and MS declare no conflict of interest.

## Supplementary information

Supplemental materials
