# Supplementary material for: The clinical effectiveness of using a predictive algorithm to guide antidepressant treatment in primary care (PReDicT): an open-label, randomised controlled trial
Source: Neuropsychopharmacology. 2021 Feb 26;46(7):1307–14. doi: 10.1038/s41386-021-00981-z (PMC8134561; doi:10.1038/s41386-021-00981-z)
Supplement: Supplementary file 1 — Supplemental materials [file 41386_2021_981_MOESM1_ESM.docx]

The Clinical Effectiveness of Using a Predictive Algorithm to Guide Antidepressant Treatment in Primary Care (PReDicT): an open-label, randomised controlled trial

Supplementary Materials

**Contents:**

1. Clinical context from which patients were recruited
2. Detailed description of exploratory analysis
3. Effect of feedback from the PReDicT test on prescribing behaviour
4. Performance of predictive algorithm
5. Detailed consort diagram by country
6. Sensitivity analysis excluding patients before update of predict test
7. Effect of country on primary analysis

**Clinical context from which patients were recruited**

Patients were recruited from clinical services in the five countries. The clinical context from which recruitment occurred and in which treatment was administered reflected the different health services. In the UK we recruited patients from 26 primary care practices throughout England, in Spain we recruited patients from 9 primary care practices and 2 secondary care practices clustered around Barcelona, in Germany we recruited patients from 8 primary care and 9 secondary care practices clustered around Würzburg and Frankfurt, in the Netherlands we recruited patients from 11 primary care and 2 secondary care services clustered around Amsterdam and in France we recruited patients from a single secondary care centre in Paris.

**Detailed Description of the Exploratory Analysis**

Analysis of the prespecified additional outcomes found an effect of using the PReDicT test on anxiety at week 8, measured using the GAD-7 questionnaire and on functional outcome at month 6, measured using the SAS-SR screener questionnaire. This raises the possibility that the early improvement in anxiety symptoms led to the later improvement in functional outcome. Statistically this proposition can be tested using a mediation analysis, that assesses the degree to which the effect of group membership on functional outcome can be explained by an indirect effect of group membership on week 8 anxiety which then impacts functional outcome (see Figure S1 below).

A 2 level path analysis model was fitted using Mplus 8.4. Baseline measures from the GAD-7 and SAS-SR questionnaires were used to correct the week 8 and 24 scores respectively. The estimated path coefficients (standard error, se) are summarised in figure S1. The overall mediation effect (se) was 0.47(0.24),p=0.048.


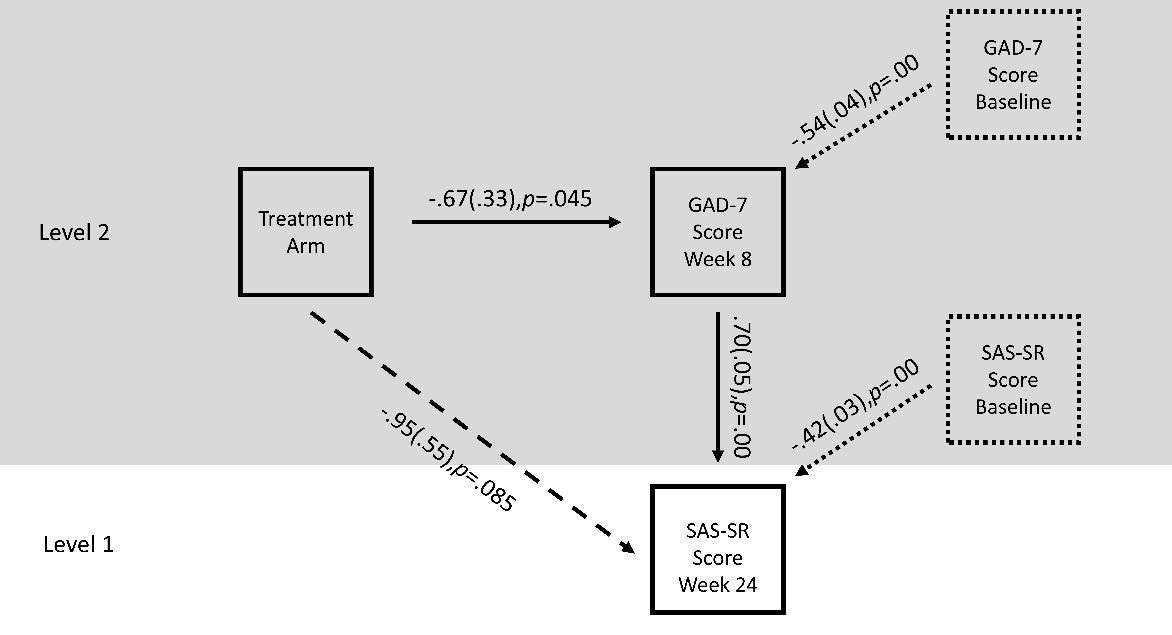


Figure S1: Variables included in the mediation path analysis. Solid boxes include the key measures included in the analysis. Solid lines represent the components of the indirect effects from treatment arm to week 8 GAD-7 and from week 8 GAD-7 to week 24 SAS-SR. Dashed line represents the direct effect of treatment arm on SAS-SR score having controlled for the indirect effect. Dotted lines and boxes illustrate baseline correction. Level 2 of the 2 level analysis is shaded in grey.

**Effect of feedback from the PReDicT test on prescribing behaviour**

In the reported study, the PReDicT test was used to prompt clinicians to amend the antidepressant treatment of those patients who were predicted to not be responding. However, clinicians were not required to change a patient’s medication, but rather were asked to consider it alongside other standard clinical variables such as patient preference, the presence of side effects etc. One limiting factor on the efficacy of using the PReDicT test is therefore the degree to which clinicians were willing to use it to amend their normal prescribing behaviour. We provide summary data on this process below. Figure S2 illustrate the proportion of patients who had the dose of their antidepressant increased (S2a), the antidepressant changed (S2b), or augmented (S2c) and the cumulative proportion of patients who had any change to their treatment (S2d) across the eight weeks of the study. The data is split by both treatment arm (PReDicT and TaU) and by the PReDicT test prediction at week 1 (i.e. predicted to be responding or predicted not to be responding). We highlight some important aspects of this data:

1. About 65% of patients in the PReDicT arm, who were predicted not to be responding, had their treatment changed within the first two weeks of the study, this compares with a rate of 10-20% in the other groups (Figure S2d). This illustrates that, as intended, clinicians used the information from the PReDicT test to amend the treatment of patients.
2. A sizable proportion of patients (35%) in the PReDicT arm, who were predicted not to be responding, did not have their treatment changed (Figure S2d). Thus clinicians did not base their prescribing decisions solely on the results of the PReDicT test. While this behaviour is consistent with appropriate clinical decision making, it does place a limit on the potential efficacy of using the test.
3. By far the most common change made to treatment was an increase in the dose of antidepressant used (Figure S2a) with almost 50% of patients in the predict arm, who were predicted not to be responding, having this change after the first week of treatment. A much smaller proportion of patients had their medication changed in the first week (7%; Figure S2b) and participants in this group had their medication augmented (Figure S2c).
4. The most substantial impact of the PReDicT test feedback was to prompt a change in treatment for those patients predicted to not be responding (blue line). The impact of a prediction of “responding” (red line), which could have prompted clinicians to stick with prescribed treatment, only slightly reduced the proportion of patients who had their treatment changed (Figure S2d).


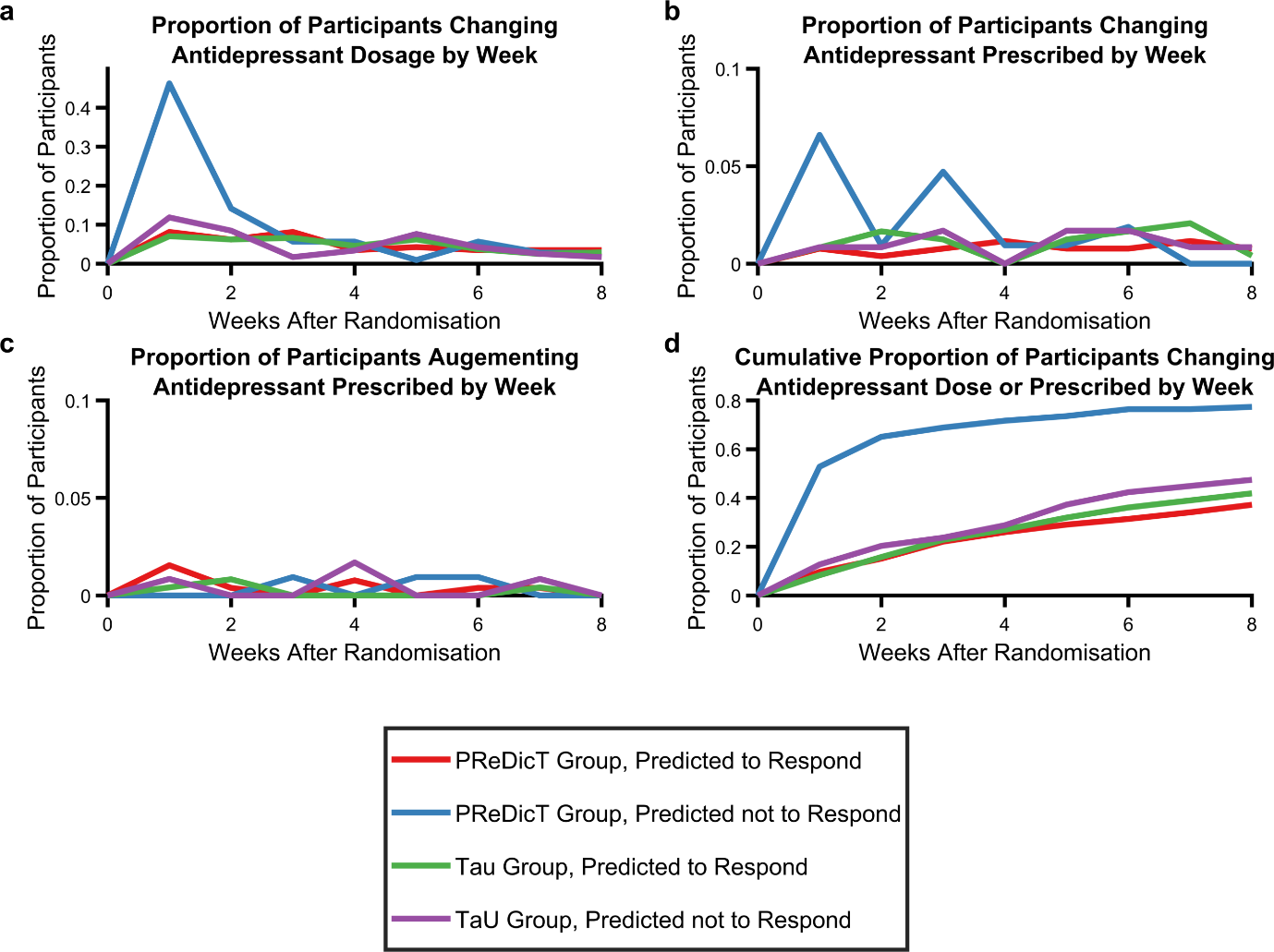


Figure S2: Effect of group membership and PReDicT test prediction on prescribing behaviour. a) the proportion of patients who had the dose of their antidepressant changed in each week of the study. b) The proportion of patients who had the antidepressant prescribed changed in each week of treatment. c) The proportion of patients who had their antidepressant augmented in each week of treatment. d) The cumulative proportion of patients who had a change made (in dose, antidepressant prescribed, or by augmentation). Proportions are presented separately for patients in the PReDicT and TaU arm (NB PReDicT test results were fed back to clinicians for patients in the PReDicT arm but not for patients in the TaU arm) and for patients in which the PReDicT test prediction at week 1 was “responding” vs. “not-responding” (a prediction of “not-responding” should prompt a change in prescriptions).

**Performance of predictive algorithm**

The development of the predictive algorithm is described in detail in our previous publication^1^. Here we provide further information on the performance of the algorithm in the current dataset. As the output of the algorithm was used to alter treatment in the PReDicT arm of the study we used data only from the TaU arm to assess algorithm performance. The overall accuracy of the algorithm was 57.5%, similar to that reported previously^1^. As can be seen from figure S3 the algorithm was more accurate when classifying responders (73% accuracy) than non-responders (40%). The predictive value of the two classes was balanced with participants having roughly 57% chance of experiencing the outcome predicted by the classifier. A summary of the performance of the classifier split by country is presented in Figure S4


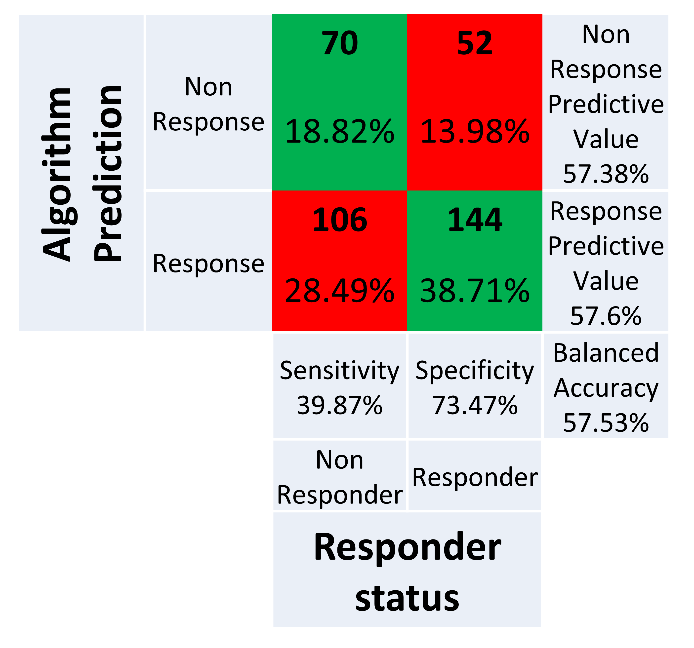


Figure S3: Predictive performance of the algorithm in the TaU group. Green cells indicate correctly labelled patients, red cells incorrectly labelled patients. Summary statistics including test sensitivity, specificity and predictive value are displayed in grey.


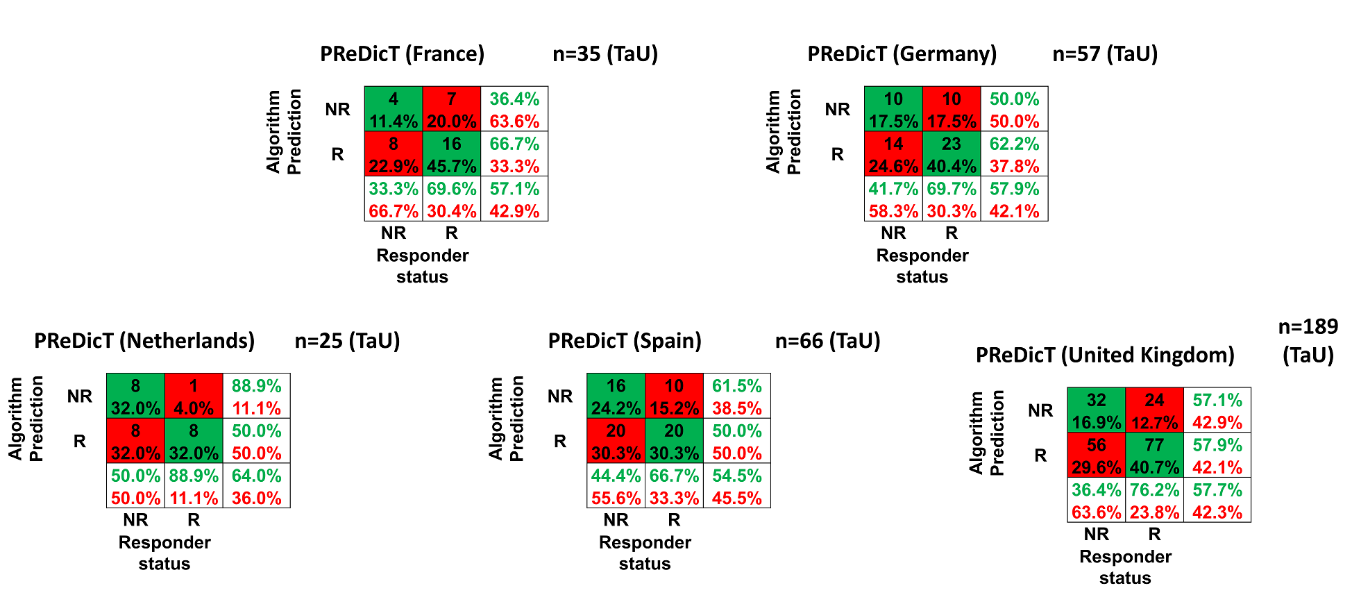


Figure S4: Predictive performance of the algorithm in the TaU group, split by country. The presented results are as described for Figure S3.

**Detailed consort diagram by country**

The consort diagram included in the main paper presents the overall recruitment and retention for the study. Figure S5 below provides this information broken down by country.


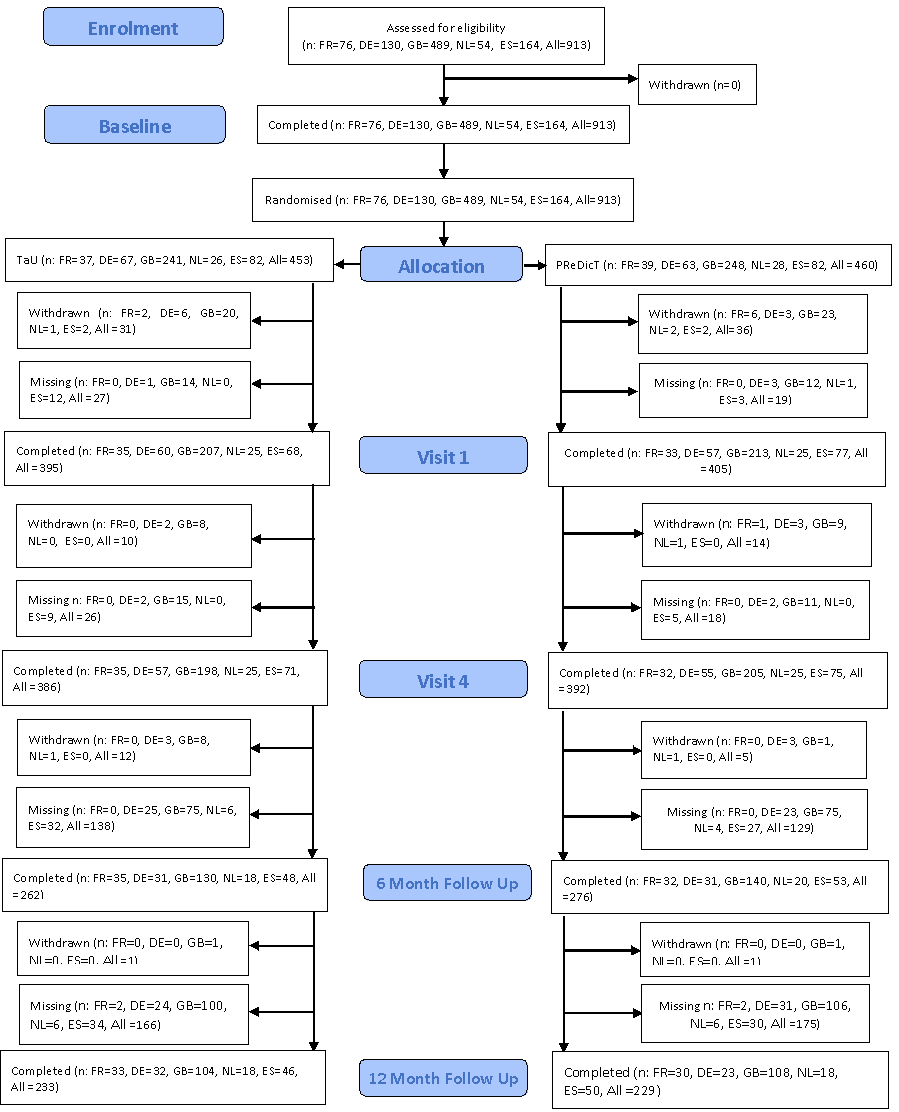


**Sensitivity analysis excluding patients before update of predict test**

As described in the methods section of the main paper, the PReDicT algorithm was updated based on the initial 67 patients recruited to the TaU arm. The updated algorithm was implemented after 155 patients had been recruited. In Table S1 below we report the statistical analyses for the primary outcome and the significant additional outcomes in both the full sample and those patients recruited following the algorithm update. As can be seen, the effect of group on the primary outcome is non-significant in both samples. In the smaller sample, the effect of the group on week 8 anxiety is no longer significant, whereas the effect on week 24 functional outcome remains significant.

Table S1: Summary of the analysis of the primary outcome and of significant outcomes from main analysis performed across the whole sample of patients and the subsample who completed the study following update of the algorithm.

| **Outcome** | **Effect Estimate (85% CI) for full sample** | **Effect estimate (95% CI) for sample recruited after algorithm update** |
| --- | --- | --- |
| **QIDS response (Odds Ration)** | 1.18 (0.89, 1.56), p=0.25 | 1.06(0.77, 1.46), p=0.712 |
| **GAD change (Mean Difference)** | 0.68 (0.03, 1.32), p=0.04 | 0.51(-0.19,1.22), p=0.152 |
| **SAS change (Mean Difference) week 24** | 2.22 (0.74, 3.70), p=0.004 | 1.82(0.11, 3.53), p=0.037 |

QIDS-SR-16; Quick Inventory of Depressive Symptoms, 16 item self-report version. GAD-7; Generalised Anxiety Disorder Assessment, seven-item version. SAS-SR; Social Adjustment Scale, self-report screener form, T-score (note a higher score indicates greater impairment).

**Effect of country on primary analysis**

The primary analysis reported in the paper included data collected from a variety of healthcare systems across five European countries. For completeness, the response rates for each country are summarised in Table S2 below.

Table S2. Response rates at week 8 in the individual countries involved in the study

| **Nation** | **PReDicT Arm** | **TaU Arm** | **PReDicT Arm** | **TaU Arm** | **Total** | **Total** |
| --- | --- | --- | --- | --- | --- | --- |
|  | *%* | *%* | *n* | *N* | *%* | *n* |
| **France** | 65.63 | 65.71 | 21/32 | 23/35 | 65.67 | 44/67 |
| **Germany** | 56.36 | 59.32 | 31/55 | 35/59 | 57.89 | 66/114 |
| **The Netherlands** | 44.00 | 36.00 | 11/25 | 9/25 | 40 | 20/50 |
| **Spain** | 49.33 | 45.07 | 37/75 | 32/71 | 47.26 | 69/146 |
| **UK** | 59.51 | 53.27 | 122/205 | 106/199 | 56.43 | 228/404 |

**Antidepressants prescribed by country**

In the study, clinicians were able to choose the antidepressant prescribed to patients. Table S3 below summarises the antidepressant medication patients were initiated on, split by country.

Table S3. Antidepressant prescription per country.

|  | **Germany** | **Spain** | **France** | **Great Britain** | **The Netherlands** | **Total Receiving each Medication** |
| --- | --- | --- | --- | --- | --- | --- |
|  | n (%) | n (%) | n (%) | n (%) | n (%) | n (%) |
| *SSRIs* | | | | | | |
| **Sertraline** | 35 (27%) | 51 (31%) | 41 (54%) | 255 (52%) | 9 (17%) | 391 (43%) |
| **Citalopram** | 32 (25%) | 71 (43%) | 1 (1%) | 213 (44%) | 26 (48%) | 343 (38%) |
| **Escitalopram** | 40 (31%) | 5 (3%) | 13 (17%) | 10 (2%) | 5 (9%) | 73 (8%) |
| **Paroxetine** | 1 (1%) | 35 (21%) | 7 (9%) | 6 (1%) | 8 (15%) | 57 (6%) |
| **Fluvoxamine** | 0 (0%) | 0 (0%) | 1 (1%) | 0 (0%) | 0 (0%) | 1 (0%) |
| **Fluoxetine^a^** | 0 (0%) | 0 (0%) | 0 (0%) | 1 (0%) | 0 (0%) | 1 (0%) |
| *Non-SSRIs^b^* | | | | | | |
| **Other (non-SSRI)** | 20 (15%) | 1 (1%) | 11 (14%) | 2 (0%) | 6 (11%) | 40 (4%) |
| *No Medication^c^* | | | | | | |
| **No medication (patient withdrawn from study before prescription)** | 2 (2%) | 1 (1%) | 2 (3%) | 2 (0%) | (0%) | 7 (1%) |
|  | | | | | | |
| **Total** | 130 (100%) | 164 (100%) | 76 (100%) | 489 (100%) | 54 (100%) | 913 (100%) |

^a^ Clinicians were asked not to initiate patients on fluoxetine in the study. Three patients from the UK were prescribed it in error. ^b^ The non-SSRI medications prescribed to patients included a large number of different medications each prescribed to a small number of patients so are not listed separately. ^c^ Patients who took no medication withdrew from the study before receiving their first prescription.

Supplementary References

1 Browning M, Kingslake J, Dourish CT, Goodwin GM, Harmer CJ, Dawson GR. Predicting treatment response to antidepressant medication using early changes in emotional processing. *Eur Neuropsychopharmacol* 2019; **29**: 66–75.
